# Onset of main Phanerozoic marine radiation sparked by emerging Mid Ordovician icehouse

**DOI:** 10.1038/srep18884

**Published:** 2016-01-06

**Authors:** Christian M. Ø. Rasmussen, Clemens V. Ullmann, Kristian G. Jakobsen, Anders Lindskog, Jesper Hansen, Thomas Hansen, Mats E. Eriksson, Andrei Dronov, Robert Frei, Christoph Korte, Arne T. Nielsen, David A.T. Harper

**Affiliations:** 1 Natural History Museum of Denmark, University of Copenhagen, Øster Voldgade 5-7, 1350 Copenhagen K, Denmark; 2 Department of Geology, Lund University, Sölvegatan 12, S-223 62 Lund, Sweden; 3 Center for Macroecology, Evolution and Climate, University of Copenhagen; 4 Department of Geosciences and Natural Resource Management, University of Copenhagen, Øster Voldgade 10, 1350 Copenhagen K, Denmark; 5 Camborne School of Mines, College of Engineering, Mathematics and Physical Sciences, University of Exeter, Penryn Campus, Penryn, Cornwall TR10 9FE, U.K; 6 Ministry of Mineral Resources, Maneq 1A, 201, P.O. Box 930, 3900 Nuuk, Greenland; 7 Akvaplan-Niva, High North Research Centre 9296 Tromsø, Norway; 8 Geological Institute of the Russian Academy of Sciences, Moscow, Russian Federation; 9 Nordic Center for Earth Evolution (NordCEE), University of Copenhagen; 10 Palaeoecosystems Group, Department of Earth Sciences, Durham University, Durham DH1 3LE, UK

## Abstract

The Great Ordovician Biodiversification Event (GOBE) was the most rapid and sustained increase in marine Phanerozoic biodiversity. What generated this biotic response across Palaeozoic seascapes is a matter of debate; several intrinsic and extrinsic drivers have been suggested. One is Ordovician climate, which in recent years has undergone a paradigm shift from a text-book example of an extended greenhouse to an interval with transient cooling intervals – at least during the Late Ordovician. Here, we show the first unambiguous evidence for a sudden Mid Ordovician icehouse, comparable in magnitude to the Quaternary glaciations. We further demonstrate the initiation of this icehouse to coincide with the onset of the GOBE. This finding is based on both abiotic and biotic proxies obtained from the most comprehensive geochemical and palaeobiological dataset yet collected through this interval. We argue that the icehouse conditions increased latitudinal and bathymetrical temperature and oxygen gradients initiating an Early Palaeozoic Great Ocean Conveyor Belt. This fuelled the GOBE, as upwelling zones created new ecospace for the primary producers. A subsequent rise in δ^13^C ratios known as the Middle Darriwilian Isotopic Carbon Excursion (MDICE) may reflect a global response to increased bioproductivity encouraged by the onset of the GOBE.

The classic studies of Sepkoski^1^,^2^ on Phanerozoic biodiversity change indicated a major increase in biodiversity during the Mid Ordovician – a conclusion supported by the development of more recent databases and sophisticated investigative tools although the inferred amplitude of the diversity spike varies^3^,^4^,^5^. All studies strongly indicate a prolonged Early Palaeozoic radiation that markedly changed the composition and structure of Phanerozoic seascapes (Fig. 1).

The main radiation of the GOBE is generally agreed to have occurred during the Mid Ordovician Darriwilian Stage^6^,^7^. Both dominant benthic and planktonic fossil groups show the same diversity pattern^8^,^9^. These often display a two-phased rise in diversity with an onset in the late Floian–early Dapingian and a second main spike during the mid Darriwilian. These marked peaks consolidated a more gradual rise in species richness within some groups, notably the phytoplankton, which was initiated during the late Cambrian^10^.

What caused the GOBE has been intensively debated^6^,^11^,^12^,^13^,^14^,^15^. Suggested drivers include both intrinsic and extrinsic factors, including increased complexity in the food web, a cooling climate perhaps driven by continental arc collisions, and even an extra-terrestrial spur to life. A number of these studies suggested a link with global cooling although palaeoclimatic data supporting cooler climate during the onset of the GOBE have been lacking; these data were either model driven with limited ground truthing or they have been questioned due to potentially flawed analytical techniques^16^. Here, we demonstrate that there is a remarkable coherence between abiotic and biotic proxies – almost bed-by-bed – which show clear evidence that a cooling indeed took place and that the GOBE seems to track this temperature decrease.

## Geological setting and location of study area

**Figure 2:**
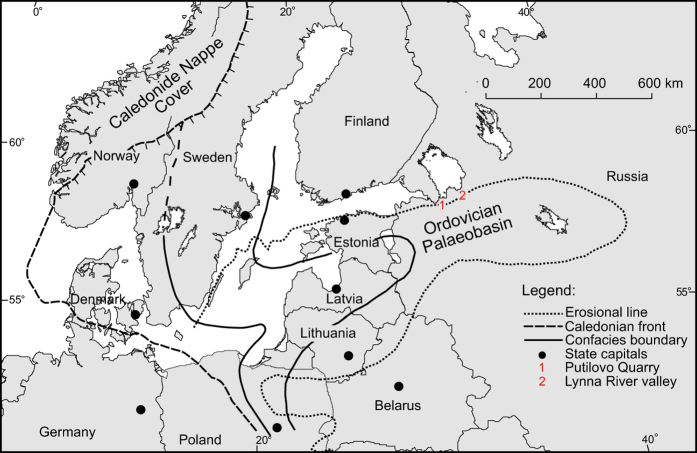
Facies belts of Baltica during the Mid Ordovician with location of study sites indicated. 1, Putilovo Quarry; 2, Lynna River valley. See legend for explanation of main geographical and geological features. Facies belts are shallowing towards the East. The map is modified from Hints & Harper (2003)^56^ with permission.

The Ordovician Period was characterized by a rapid, northward drift of several palaeoplates that had rifted off Gondwana^17^. This intense plate tectonic activity resulted in an extreme first-order sea level rise that potentially culminated in a Phanerozoic sea level maximum in the Late Ordovician^18^,^19^ generating widespread epicontinental seas which have no modern analogues. One of these extensive seas was established across large parts of the palaeocontinent of Baltica (Fig. 2). During the Early–Mid Ordovician, Baltica moved rapidly from the cool temperate climate zone at about 50 °S towards warmer temperate latitudes, reaching about 40 °S in the mid Darriwilian^17^. Thus, the more shallow-water facies of this palaeobasin is characterized by cool-water carbonates^20^. Overall, the depositional facies changed westwards in Baltoscandia from nearshore, detritic wacke–grainstones intercalated with marls, through finer grained limestones without marl interbeds to offshore graptolitic shales in the deepest parts of the palaeobasin. Overall, siliciclastic input was extremely limited.

The upper Lower to Middle Ordovician (Floian–Darriwilian) succession was intensively sampled bed-by-bed during several field campaigns in the St. Petersburg area, Russia and northern Estonia. The main outcome has been a very large palaeobiological dataset that precisely pinpoints the initiation of the GOBE based on a study of more than 30,000 rhynchonelliformean brachiopods^21^. Together with 15,000 trilobites, this dataset provides an exceptionally detailed mid-latitude palaeoecological window into the biotic changes on the palaeocontinent of Baltica at this time. In addition, we compiled a geochemical dataset with stable isotope and trace element data for each bed in the succession, based on more than 200 brachiopod shells.

The setting on the interior of a large stable craton provides an ideal laboratory for the study of sea level changes during the Ordovician, as local depth was little affected by tectonic disturbances. The entire Ordovician succession is condensed with a total thickness of less than 100 m in the study area. Net depositional rates were extremely low with average sedimentation rates of less than 2 mm per 1000 years and thus the effect on local water depth and, with that, on seawater temperature was minor. Furthermore, the craton was deeply peneplaned and characterized by an exceptionally low relief 20. In this type of depositional setting even small fluctuations in the eustatic sea level predisposed vast areas to either exposure or flooding. At the same time, the basin had a wide, ocean-facing gateway encouraging the circulation of water across the craton. This is confirmed by the fauna representing fully marine conditions supporting euhaline conditions. In addition, due to limited burial, the region has been little affected by subsequent diagenesis which is corroborated by pristine fossil preservation as displayed by elemental distribution and the well-preserved ultrastructure of the analyzed brachiopod shells (Supplementary Information and Supplementary Figs 1–3).

**Figure 3:**
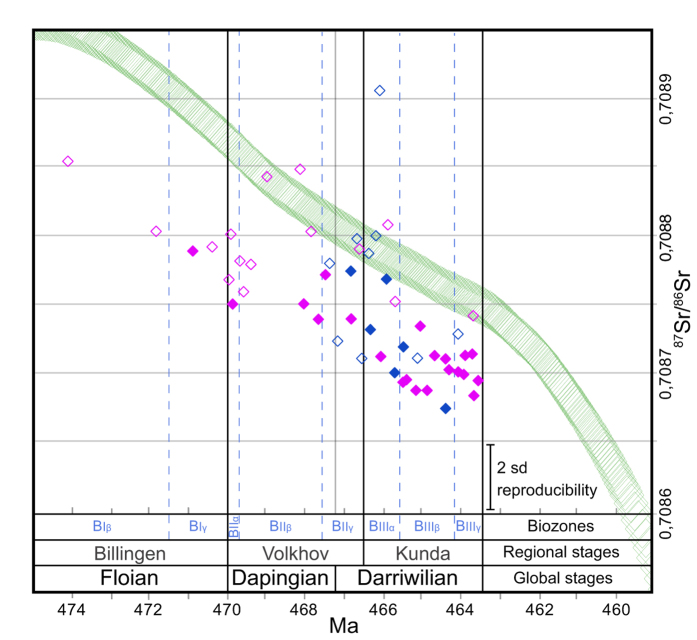
^87^Sr/^86^Sr Strontium ratios plotted against stratigraphy based on 51 brachiopod specimens from the two studied sections. Inferred global trend based on the LOWESS fitted line^23^ (marked in green) is included for comparison. Solid pink rhombs indicate Putilovo Quarry; blue, Lynna River valley. Open rhombs represent samples with trace element composition above the operational preservation limit applied. Thus, these are regarded as less trustworthy. Error bar on the reference material is shown in the lower right corner. See Supplementary Information file for details of uncertainties in the analyses.

We focused on two sections, Putilovo Quarry and the Lynna River valley, which are located 80 km apart in western Russia (Fig. 2; Supp. Figs 1 and 2). The sections are correlated using macrofossils and fluctuations in the geochemical signals are also easily tracked in the respective sections. Global correlations of the sections are further confirmed by excursions in δ^13^C_carb_^22^ and ^87^Sr/^86^Sr chemostratigraphy – the latter matching the declining Floian–Mid Darriwilian secular trend of the fitted LOWESS line^23^ (Fig. 3).

## Bed-by-bed correlation of sections

**Figure 4:**
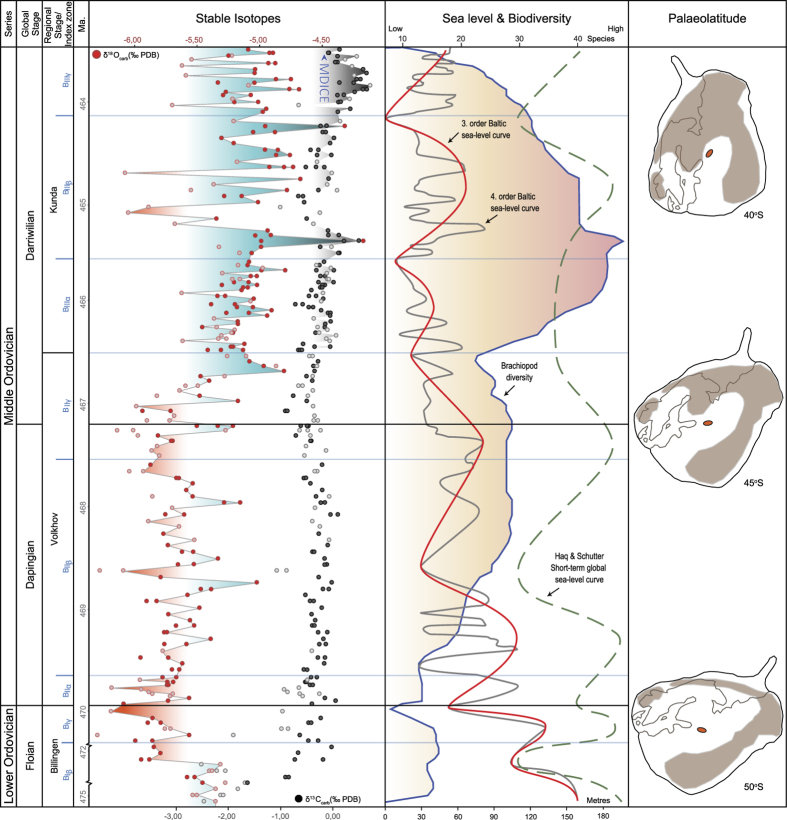
Summary figure showing bed-by-bed stable isotope geochemistry, sea level change and rhynchonelliformean brachiopod diversity^21^ through the studied interval. In addition, the palaeogeographical northward move and anti-clockwise rotation of Baltica^17^ is illustrated along with our tentative approximation of Baltoscandian land areas through the studied interval (brown shading) and the location of the study area (red oval). Solid circles in the stable isotope column denote samples below the operational preservational limit; open circles are samples above. Onset of MDICE-excursion indicated in the δ^13^C_carb_ curve. Green, punctuated line shows global, short-term fluctuations^18^. Abbreviations: Ma, million years.

The Lower-Middle Ordovician succession in Baltoscandia is divided into trilobite zones that can be correlated across the entire region. The trilobite zones have been assigned regional index numbers which are used across the eastern part of the Baltoscandian craton. The index numbers are used in the figures herein along with the regional stages, as they allow for high precision correlation of sections. We have further tied them to global stages using conodont and graptolite biostratigraphy^24^. See Supplementary Figs 1 and 2 for a schematic overview of the trilobite zones, index numbers, regional and global stages used in the current study. In Figs 3 and 4 they are further linked to chronostratigraphical ages using the most recent time scale^25^. ^87^Sr/^86^Sr ratios, analyzed specifically on 51 brachiopod shells for the current study, corroborate these age estimates. They provide the first cluster of ^87^Sr/^86^Sr ratios from the Ordovician of Baltica and thus further refine the global data through an interval that previously had little data coverage^23^. This is arguably why our data points plot a little lower than the inferred global trend represented by the LOWESS fitted line^23^ (Fig. 3).

In the East Baltic area, the horizontally stratified limestone succession is condensed. Thus, the approximately 16 m of section in the Putilovo Quarry, which form the basis for the current study, correspond to a 10–12 myr interval ranging from the Lower Ordovician Floian (Fl1, *Prioniodus elegans* conodont Zone) to the Middle Ordovician Darriwilian (Dw2, *Eoplacognathus pseudoplanus* conodont Zone) stages^22^. In Putilovo Quarry roughly 150 beds were sampled, whereas in the less condensed Lynna River valley section, which encompasses the Dapingian (Dp3) – Darriwilian (Dw2) interval, approximately 100 beds were sampled. High precision correlation was achieved by using brachiopods and trilobites, which enabled a bed by bed biostratigraphical correlation^21^,^26^,^27^,^28^,^29^,^30^,^31^. The biostratigraphical correlations were further refined by establishing biofacies which facilitated correlation within an ecostratigraphical framework^26^,^31^. Thus correlation of these sections is exceptionally well constrained.

Although the two studied sections are part of the same facies belt, they represent an oblique depth transect into the eastern part of the basin: The Putilovo section is more proximal which is most evident in the lower part of the Kunda Regional Stage (Dw2), which thins westwards from more than 3 m in the Lynna River valley section to roughly 0.5 m in the Putilovo section. This interval marks the start of the main biodiversity pulse within the shelly benthos^21^,^26^.

## Establishing statistically supported biofacies: the basis for the sea level curve. 

In order to construct a high resolution sea level curve through the sections, statistically supported biofacies were established bed-by-bed based on more than 45,000 macrofossils^26^,^31^. The data were analyzed using detrended correspondence analyses and cluster analysis in the software package PAST^32^. The precise multivariate methods applied are described in the literature^26^,^33^.

The multivariate data analyses support the establishment of a set of trilobite and brachiopod biofacies. This study operates with five depth-related biofacies of which the shallowest is based exclusively on brachiopods (Biofacies 1). This is dominated by the *Lycophoria* and *Gonambonites* associations^26^. Biofacies 2 is characterized by the trilobite genus *Asaphus* and, in the Kundan interval, also the brachiopods *Orthis* and *Orthambonites*. An intermediate biofacies dominated by the trilobite genera *Ptychopyge* (*s.l.*) and *Rhinoferus*, constitutes Biofacies 3. It is succeeded basin-wards by Biofacies 4, which is dominated by the trilobites *Megistaspis* and *Niobella*. Finally, the most offshore faunal association, which is here termed Biofacies 5, is dominated by the trilobites *Megalaspides* and *Paramegistaspis*.

## Results

We present scaled 3^rd^ and 4^th^ order sea level curves through the succession (Fig. 4; Supp. Figs 1 and 2). These are based on detailed regional bio- and lithofacies changes through the studied time interval. The biofacies outline several major sea level oscillations, during an overall regressive trend through the early Mid Ordovician^26^,^33^, including the onset of the GOBE in the early Darriwilian^21^. This regressive trend is recognized globally^18^.

### Palaeoenvironmental changes based on biofacies

The upper Floian part of the succession (*Megistaspis estonica* trilobite Zone, See Supp. Fig. S1) is exclusively characterized by the deep water Biofacies 5. This level corresponds to the already falling sea level, terminating the *evae* highstand^34^ and which ended in a transient lowstand at the Floian–Dapingian boundary. The succeeding lower part of the Dapingian Stage is dominated by Biofacies 4 and thus represents slightly shallower water conditions. Especially in the lower part of the Volkhov Regional Stage, in the *Asaphus broeggeri* Zone, the sea level fluctuated considerably, as witnessed by recurring shifts between Biofacies 4 and 2. In the upper part of the Volkhov Stage, the sea level was more stable, as signalled by a long interval characterized by Biofacies 3.

The lowermost Darriwilian (uppermost Volkhovian *Asaphus lepidurus* trilobite Zone) represents a prominent, long-lasting sea level drop characterized by Biofacies 2 (see Figs S1 and S2). In the succeeding Kunda Regional Stage, the lowermost biozone, the *Asaphus expansus* trilobite Zone, is much expanded in the Lynna River valley section, compared to that seen in Putilovo Quarry (compare the thickness of the biozones in Figs S1 and S2).

The *A. expansus* Zone commenced with *Orthis* dominated faunas, representing a small initial drowning, but still within the Biofacies 2 depth range. In the lower half of the *A*. *expansus* Zone this facies continues to dominate, although *Lycophoria* and *Gonambonites* dominated faunas become increasingly abundant upwards. These represent Biofacies 1 and signal an overall shallowing. The upper part of the *A*. *expansus* Zone and the lower part of the overlying *Asaphus raniceps* trilobite Zone is dominated by genera associated with Biofacies 1, thus signalling a major shallowing. These trends can also be recognized in Scania–Bornholm, Västergötland and the Oslo Region^33^,^35^ and this level is inferred to represent the second shallowest interval in the entire succession. It corresponds to the ‘Täljsten’ marker bed interval found in other areas of Baltoscandia^36^, but can also be tracked globally^26^.

Hereafter followed a substantial drowning, represented by the first influx of *Orthambonites* dominated faunas, which here is regarded as representing Biofacies 2. This corresponds to the Basal Llanvirn Drowning Event which is a globally recognized sea level rise^34^. Most of the remaining part of the *A*. *raniceps* - *A*. *striatus* Zone is dominated by the *Orthambonites* association, i.e. Biofacies 2. However, the topmost beds record a significant shallowing which we regard as the shallowest interval in the studied succession, nearly completely dominated by fragmented, thick *Lycophoria* shells. It is possible that a full regression took place in the area at this level. This regression is also recorded globally^18^. Finally, the *Asaphus minor* trilobite Zone represents a more mixed biofacies, overall suggestive of deepening conditions.

### Scaling the sea level curve

We have combined our sea level curve with existing Baltoscandian data^26^,^33^,^34^ in order to estimate the scale of the 3^rd^ and 4^th^ order sea level changes through the Early–Mid Ordovician (Floian–Darriwilian). The new scaled curve is based not only on the very detailed palaeoecological studies outlined above, but also on sections through offshore facies in Scania–Bornholm, intermediate facies in the Oslo Region and nearshore facies from other localities in Russia–Estonia^26^,^27^,^28^,^33^,^34^,^37^. The fact that the sections constitute a depth transect on the palaeoshelf has guided scaling of the curve, as reconstructed sea level oscillations should satisfy observed changes both in deep and shallow settings. It appears that the cool water limestone facies started to develop well below the storm wave base; the lime mud deposited in the deeper part of the shelf is assumed to have been winnowed from the shallower shelf. The main clastic supply was from the west^20^. The major sea level fall that took place in the early Mid Ordovician led to a westwards (downslope) migration of the limestone facies into deeper parts of the shelf that through most of the Ordovician was characterized by deposition of graptolite shale facies. This limestone tongue is named the Komstad Limestone in southernmost Scandinavia and the Huk Formation in southern Norway. As the sea level continued to fall, this limestone spread farther and farther west^33^,^34^,^38^. Maximum western extent is seen in the upper part of the *A. raniceps* Zone, believed to signal peak lowstand. On the inner shelf most intervals with falling sea level and all lowstands were associated with cessation of deposition but it is uncertain whether the studied Russian and Estonian sections experienced a full regression during the most major lowstand peaks. Unambiguous sedimentological evidence of subaerial exposure is lacking, but the area was probably close to or emergent during the late *A*. *raniceps* trilobite Zone. Overall, we estimate the sea level oscillations in the studied interval to be in the order of 150 m in Baltica. In comparison, global data suggest sea level fluctuations at least in the order of 80–90 m^18^, mirroring the trends in the 3^rd^ order curve produced in this study (See Fig. 4).

### Floian–Darriwilian δ^18^O fluctuations in the successions

With respect to the geochemical data, the lower part of the succession commences with heavier brachiopod δ^18^O values reflecting the intermediate palaeogeographical latitude of Baltica during the Floian (Fig. 4). In the uppermost Floian the values become more than 0.5 ‰ lighter ranging down to −6.0 ‰. The light oxygen isotope values continue during the Dapingian with baseline values fluctuating around −5.5 ‰. This trend is suddenly interrupted by a significant increase of more than 1 ‰ at the Dapingian–Darriwilian transition. The Kundan Stage continues this dramatic increase in δ^18^O values with one sample even as high as −4.2 ‰ in the lowermost *A. raniceps* Zone. This is succeeded by a fast drop down to −5.4 ‰ before it rapidly increases to −4.7 ‰. The remaining part of the *A*. *raniceps* Zone exhibits decreasing δ^18^O values although the topmost beds show dramatic increases up to −4.3 ‰. The uppermost biozone, the *A. minor* Zone, shows fluctuating values between −5.3 ‰ to −4.7 ‰.

### Floian–Darriwilian δ^13^C_carb_ correlation and the nature of the MDICE spike

The brachiopod δ^13^C_carb_ values increase from below −2.4 ‰ to around 0.0 ‰ in the Billingen interval. The pronounced lighter δ^13^C values in the lowermost beds probably correlate with the globally occurring negative excursion in the *Tetragraptus approximatus* graptolite Zone^22^. Hereafter follows a relatively stable interval with baseline values around −0.3 ‰ up through the first two biozones in the Volkhov Stage. This is succeeded by a slight decrease in baseline values in the uppermost Volkhovian *A. lepidurus* Zone. This decrease is continued into the lowermost Kundan before a sharp increase to positive values sets in in the uppermost *A*. *expansus* – lower *A*. *raniceps* zones. The more expanded Lynna section indicates a two-phased increase in the *A*. *expansus* interval (Fig. S2). Succeeding the peak in the lower *A*. *raniceps* Zone a rapid drop back to negative values (−0.7 ‰) follows. This is followed by a steady, continued increase up through the remaining part of the section with a peak around 0.7 ‰. This positive δ^13^C trend towards the top of the succession clearly corresponds to the initial part of the globally occurring Middle Darriwilian Isotopic Carbon Excursion (MDICE)^22^,^39^. This has previously been indirectly related to glaciation^40^. Here we show, however, that the marked cooling started several million years prior to the main spike in the MDICE (not covered by the present study). We propose that this increase in the δ^13^C_carb_ values in the lower Darriwilian was generated by an increase in bioproductivity caused by the rapidly accelerating radiation. Thus, the MDICE is the global response to the ignition of the GOBE.

## Discussion

Apart from the decoupling of the sea level curve and the oxygen isotope ratios in the lowermost beds studied (Billingen interval), the palaeoecologically derived 3^rd^ and 4^th^ order sea level curves and the δ^18^O curves mirror each other throughout the studied interval; that is, heavier δ^18^O values track falling sea level almost on a bed to bed scale. In the uppermost Billingen interval a short warming event has been demonstrated by the influx of tropical conodonts into Baltica^41^. This warming event is captured by the δ^18^O isotopes showing a rapid drop of more than 0.5 ‰. This suggests that the heavier δ^18^O values below indicate that Baltica was positioned at sufficiently high southerly latitudes to generate such high δ^18^O values. As for the peak lowstand interval in the Kundan *A*. *expansus*–*A*. *raniceps* interval, this correlates with the highest values in the δ^18^O curve.

This sudden shift to heavier, strongly fluctuating δ^18^O values just after the Dapingian–Darriwilian boundary with 1 to 1.5 ‰ shifts in the Kundan interval are remarkably similar to the δ^18^O pattern observed during the Quaternary^42^,^43^. Accepting that no major salinity changes took place in this well ventilated, mid-latitude palaeo-basin, this oxygen isotope increase is suggestive of a 4 to 5 °C cooling^44^. The counterclockwise rotation of Baltica^17^ at this time could theoretically have contributed to this shift as the ocean gateway turns increasingly towards the south, theoretically permitting cooler ocean currents onto the platform (Fig. 4). However, in that case, a more gradual shift to heavier oxygen isotopes would be expected. Further, the palaeoecological proxies indeed support a pronounced sea level fall controlled by glacioeustasy as witnessed by the rapid shifts in biofacies (Supp. Figs 1 and 2). An overall 4–5 °C decrease across the Dapingian–Darriwilian transition, indicated by data from benthic fossils, thus probably represents a temperature shift at the sea floor (see discussion of assumptions in the Supplementary Information file). This is a conservative estimate because Baltica drifted more than 1,000 km closer to the Equator through the studied interval^17^. This continued northward drift towards warmer, mid-latitude climate belts, ought to some extent have counter-acted the cooling climate. However, our two separate proxies suggest an opposing climate signal. Similarly, because of the regressional trend we would expect warmer water across the craton, but the opposite is true. We conclude that the observed fall in sea bed temperature represents a major ocean cooling event and a shift from greenhouse to icehouse causing a major glacioeustatic sea level drop. We estimate the sea level fall to have been at least 150 m from the global upper Floian *evae* highstand^18^,^34^ to the most shallow water facies in the uppermost *raniceps* Zone in the lower Darriwilian. This is in the same order of magnitude as estimates for the Pleistocene sea level fluctuations^45^. We argue that only glacioeustatic oscillations could have enabled the large sea level drop through the studied succession. The cooling and rapidly fluctuating sea level reflects the waxing and waning of ice caps at the palaeo South Pole. Thus, we suggest an onset of Ordovician icehouse conditions which commenced around the Lower/Middle Ordovician boundary, significantly changing the traditional view of the Ordovician as a sustained greenhouse interval.

What caused this sudden cooling is not resolved by the current study. Previously suggested drivers for the general cooling climate trend in the Ordovician^6^,^11^ are mostly based on gradual processes, such as continental arc collisions^14^ or the colonisation of land plants^11^. These are drivers that are primarily inferred from a record of reduced weathering, as witnessed by the declining ^87^Sr/^86^Sr record through the Ordovician^23^. This, again, is interpreted to reflect a slow decline of CO_2_ levels. This scenario is incompatible with the current study as these drivers probably did not contribute to the sudden shift in climate demonstrated by the present study.

The sudden initiation of icehouse conditions during the Mid Ordovician thus represents a paradigm shift in the way we view Ordovician climate. The onset of the greatest radiation event of the Phanerozoic may now be addressed against a background of a sudden shift in climate. This would have had a profound impact as cooler ocean currents increased thermohaline circulation in the oceans, creating upwelling zones where plankton evolved and radiated. Moreover, oxygen was more accessible for organisms, once ocean temperatures fell, as predicted by Henry’s Law.

**Figure 5:**
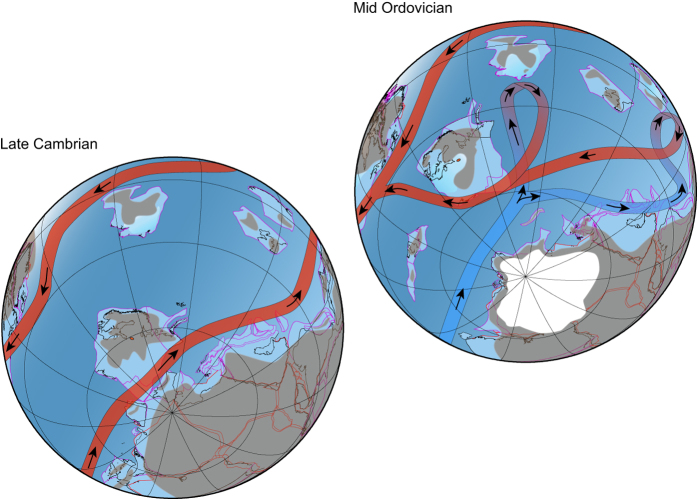
Simplified hypothetical ocean conveyor belt circulation scenarios for the late Cambrian and Mid Ordovician worlds. During the late Cambrian greenhouse, latitudinal temperature gradients were weak resulting in warm surface water circulation, but lacked movement of deep-water masses. This resulted in widespread euxinia at the sea floor. The onset of icehouse conditions during the Mid Ordovician intensified the latitudinal and bathymetrical temperature and oxygen gradients. This initiated the formation of deep ocean circulation and thus the Great Ocean Conveyor Belt. Ocean currents are based on the literature^49^,^50^,^51^,^52^,^53^,^54^,^55^, but inferred based on the circulation pattern of the present day Great Ocean Conveyor Belt. Palaeogeographic projection provided by Trond Torsvik, Norwegian Geological Survey, based on the software package BugPlates^57^: http://www.geodynamics.no/Web/Content/Software/.

The onset of the Mid Ordovician icehouse marked a dramatic climatic change, contrasting conditions in the late Cambrian world. This clearly had a fundamental impact on ocean circulation patterns (Fig. 5). Widespread black shales suggest that anoxia was prevalent on late Cambrian ocean floors and that water circulation was at best sluggish^46^,^47^. During the Ordovician, the increased latitudinal temperature gradients, as well as those for bathymetrical oxygen, encouraged by icehouse conditions initiated thermohaline circulation bringing nutrient rich, cold bottom waters to the surface. This effect was further intensified by a drastically rising sea level, which flooded the continents, creating epicontinental seas that were shallow and thus more easily heated. In addition, the continued northbound drift of several continents and microcontinents towards lower latitudes further intensified the thermohaline circulation through the Ordovician as more and more shelf areas impinged on lower latitudes transporting larger areas with upwelling zones into tropical regions.

We speculate, therefore, that the initiation of a Lower Palaeozoic Great Ocean Conveyor Belt encouraged the GOBE by creating new viable ecospace in the form of nutrient rich upwelling zones. This initiated a revolution not only for the primary producers, but for the entire trophic chain.

## Methods

Our dataset consists of ~30,000 brachiopods and 15,000 trilobites collected bed-by-bed from several localities in the eastern Baltic–western Russian region along a 400 km transect. All specimens were prepared mechanically using air chisels and registered in a database. Specimens were identified at the lowest possible taxonomical level. Sections were correlated using bio- and lithostratigraphy, as well as ecostratigraphy for the upper Volkhovian–Kundan interval^26^. For the current study, an estimate of the absolute sea level changes through the studied interval were conducted based on compilation of regional sedimentological and palaeontological data. The presented stable isotope data originate from two of these sections, which are located 80 km apart in western Russia. Calcite for stable isotope analysis was carefully extracted from the secondary layer of the brachiopod shells, as this part of the brachiopod shell is most likely to carry intact geochemical information (see Supplementary Information). One shell was selected from each bed in the two studied sections. Up to three samples were taken from each shell if possible. In one specimen seven samples were taken to study the expected seasonal variation. Thus, in all, the dataset comprises 385 samples obtained from 234 shells. To avoid using material that had been subjected to secondary diagenetic overprint, all samples were photographed under SEM to visually screen for ultrastructure signs of diagenetic alteration. Further, trace element analyses were conducted to study Mn/Ca, Mg/Ca and Sr/Ca ratios in order to assess the preservational state of the shells analyzed. Here a limit of 250 μg/g (=0.455 mmolMn per mol Ca) was applied for Mn to indicate good preservation^48^.

## Additional Information

**How to cite this article**: Rasmussen, C. M. Ø. *et al.* Onset of main Phanerozoic marine radiation sparked by emerging Mid Ordovician icehouse. *Sci. Rep.* **6**, 18884; doi: 10.1038/srep18884 (2016).

## Supplementary Material

Supplementary Information

Supplementary Dataset

## Figures and Tables

**Figure 1:**
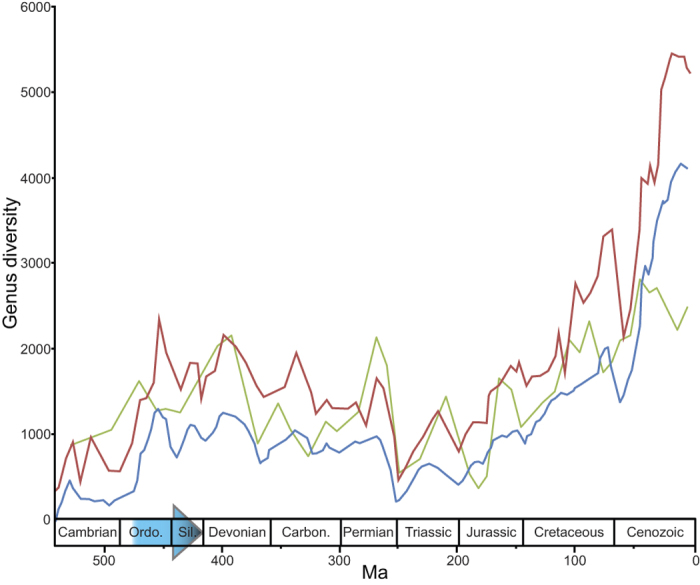
Estimates of Phanerozoic marine generic biodiversity. Red line based on Sepkoski^2^; blue line based on Rohde and Muller^5^ and green line based on Alroy *et al.*^4^. Note that the green line is not to scale (maximum number of genera approximately 500). Blue arrow indicates the main onset of the GOBE^2^,^5^,^8^,^9^, including its possible prolonged duration into the Silurian Period^4^. Sepkoski’s dataset downloaded from http://strata.geology.wisc.edu/jack/.
