# Supplementary material for: Onset of main Phanerozoic marine radiation sparked by emerging Mid Ordovician icehouse
Source: Sci Rep. 2016 Jan 6;6:18884. doi: 10.1038/srep18884 (PMC4702064; doi:10.1038/srep18884)
Supplement: Supplementary Information [file srep18884-s1.pdf]

Onset of main Phanerozoic marine radiation sparked by emerging Mid Ordovician icehouse

Supplementary Information

Christian M. Ø. Rasmussen, Clemens V. Ullmann, Kristian G. Jakobsen, Anders Lindskog, Jesper Hansen, Thomas Hansen, Mats E. Eriksson, Andrei Dronov, Robert Frei, Christoph Korte, Arne T. Nielsen, David A.T. Harper

Supplementary Information figures

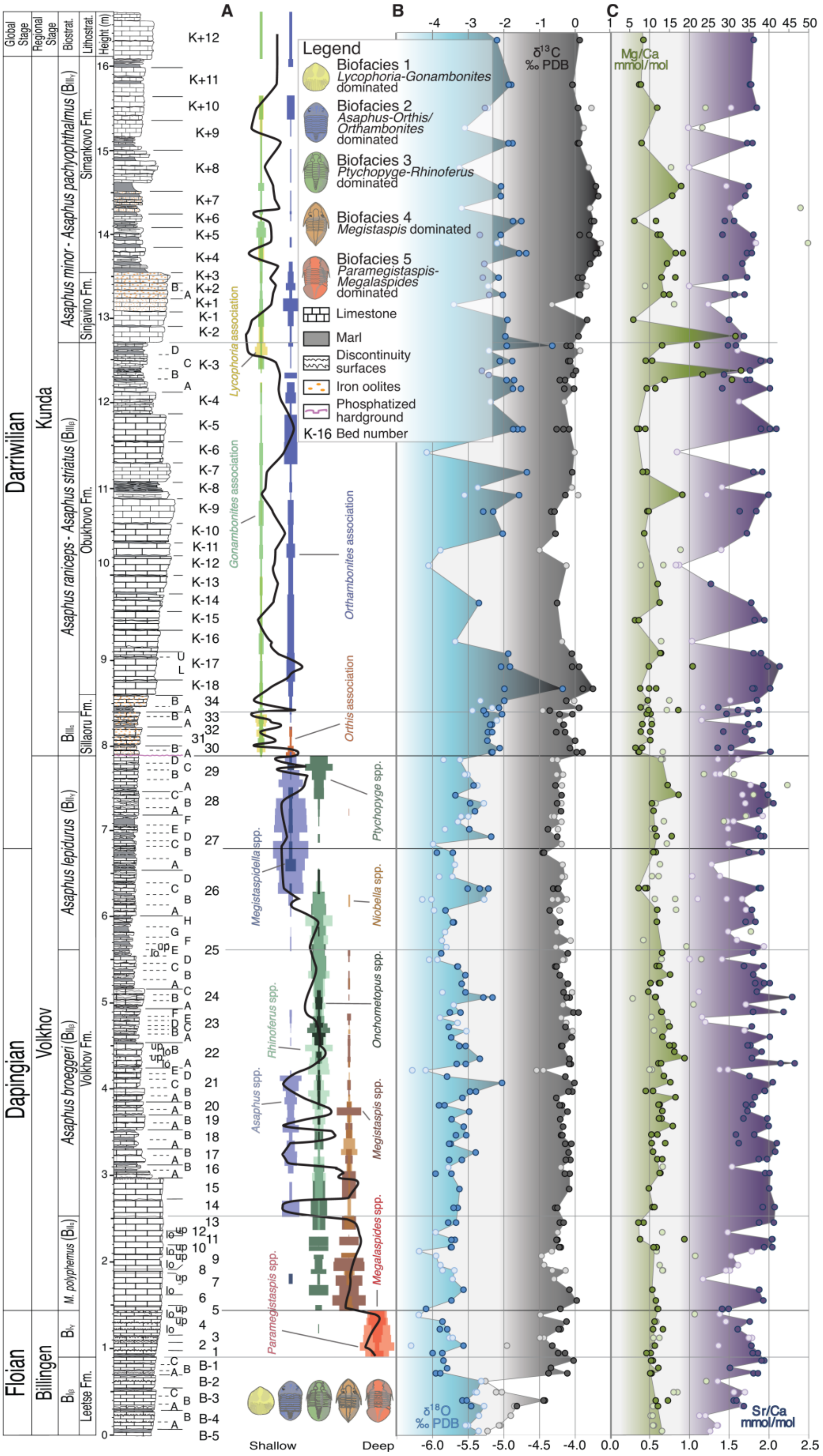

Supplementary Fig. 1 | Type log from Putilovo Quarry showing paleobiological and geochemical proxies bed by bed. The log is tied to local litho- and biostratigraphy, as well as regional and global stages. B, Relative sea level curve based on the statistically supported biofacies. Thickness of colored strokes (different shadings) bed-by-bed represent the relative dominance of the main genera, or, associations in a given biofacies. B, Brachiopod $\delta^{18}O$ and $\delta^{13}C_{carb}$ values through the succession. C, Trace element concentrations. For the geochemical proxies, samples below the operational limit are represented by solid circles, whereas samples above are indicated by open circles (see Supplementary Material for measures taken to account for secondary diagenetic overprint of the geochemical proxies). Abbreviations: Biostrat, Biostratigraphy; Lithostrat, Lithostratigraphy; $B_{I\beta}$, *Megistaspis* aff. *estonica – Megalaspides dalecarlicus* Zone; $B_{I\gamma}$, *Megistaspis estonica* Zone; $B_{II\alpha}$, *Megistaspis polyphemus* Zone; $B_{III\alpha}$, *Asaphus expansus* Zone.

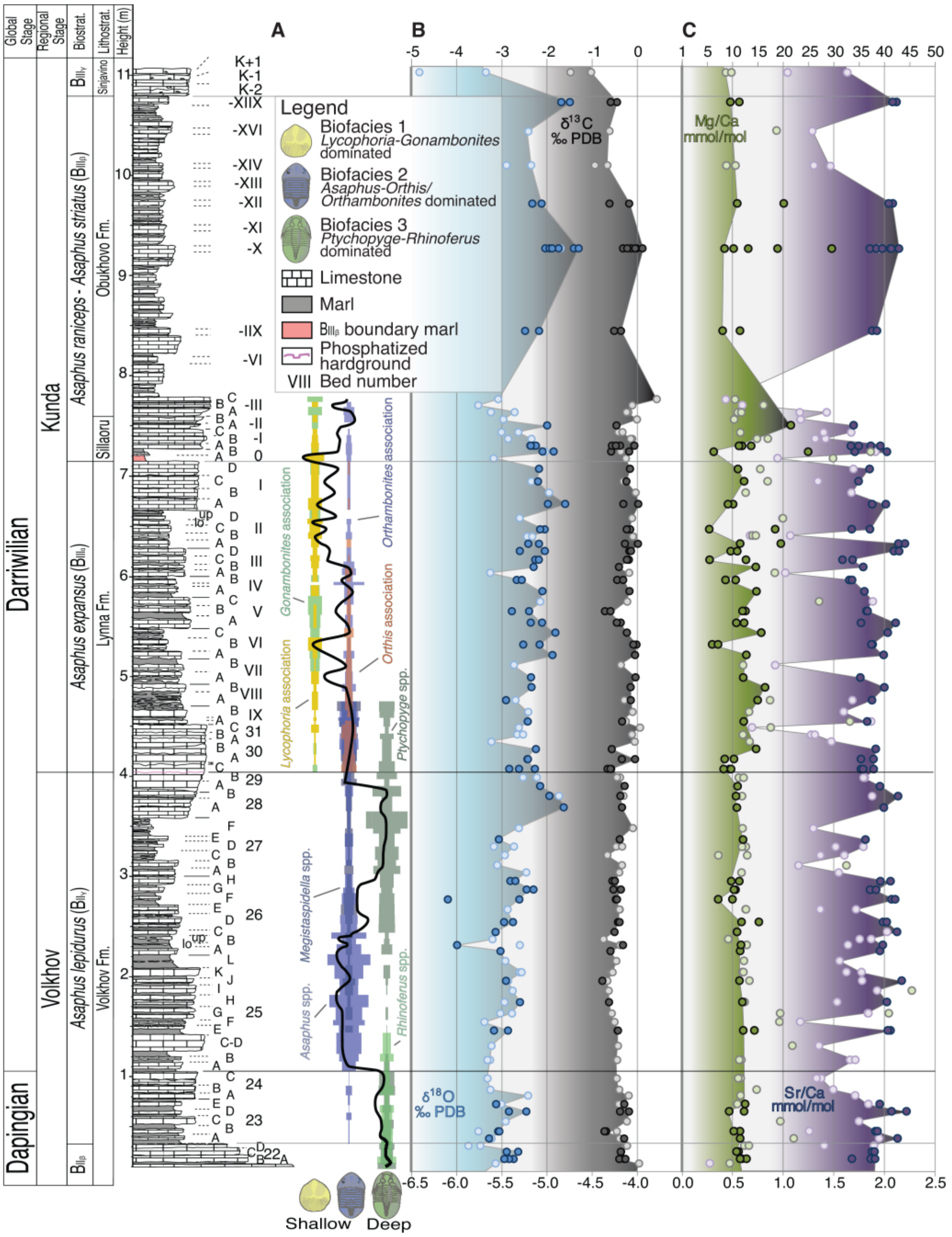

Supplementary Fig. 2 | Type log from Lynna River valley showing paleobiological and geochemical proxies bed by bed. The log is tied to local litho- and biostratigraphy, as well as regional and global stages. A, Relative sea level curve based on the statistically supported biofacies. Thickness of colored strokes (different shadings) bed-by-bed represent the relative dominance of the main genera, or, associations in a given biofacies. B, Oxygen and $\delta^{13}C_{carb}$ values through the succession. C, Trace element concentrations. Abbreviations: $B_{II\beta}$, *Asaphus broeggeri* Zone; $B_{III\gamma}$, *Asaphus minor – Asaphus pachyophthalmus* Zone.

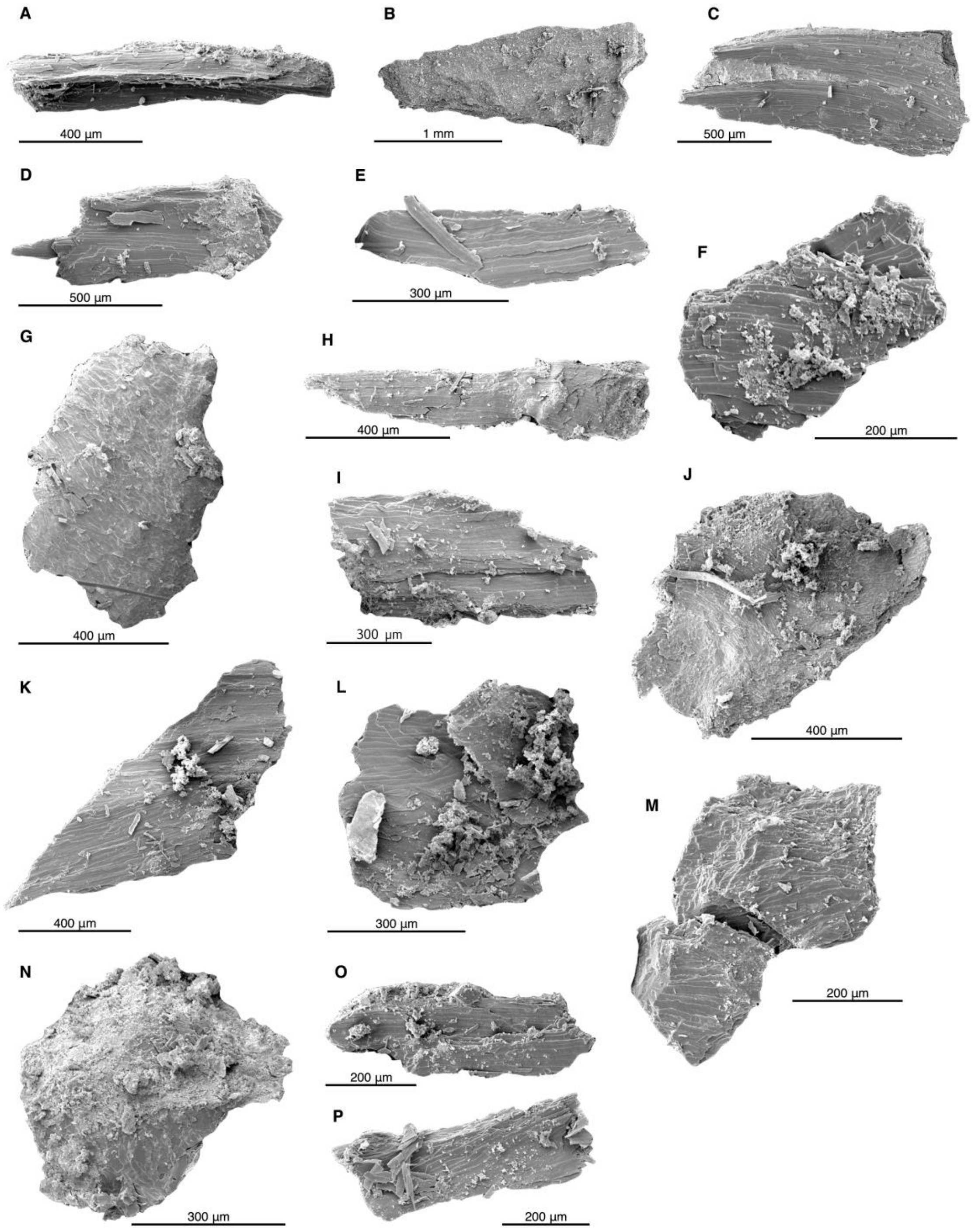

Supplementary Fig. 3 | Ultrastructure of selected brachiopod specimens below and above the operational limit applied for trace element ratios. In general, most specimens appear well preserved based on the ultrastructure with smooth surfaces and show no indication of textural changes. *Dust from the preparation by the needle is present in nearly all of the images.* T*his dust originates from parts of the well preserved secondary layers.* A – I, well

preserved specimens (limiting Mn/Ca ratio below 0.455 mmol/m) from each biozone through the succession (listed as bed number, index zone and global time slice): A, Bed 4 upper, $B_{I\gamma}$ (Fl3).B, Bed 5A, $B_{II\alpha}$(Dp1). C, Bed 21C, $B_{II\beta}$ (Dp3). D, Bed 25A, $B_{II\beta}$. E, Bed 27D, $B_{II\gamma}$. F, Bed IIIB, $B_{III\alpha}$ (Dw1). G, Bed 30B, $B_{III\alpha}$. H, Bed K-3C, $B_{III\beta}$ (Dw2).I, Bed K+4, $B_{III\gamma}$. J – P, samples (bed number and index zone listed) with trace element values in excess of the operational limit: J, Bed 5 lower, $B_{I\gamma}$. K, Bed 10 lower, $B_{II\alpha}$. L, Bed 23E, $B_{II\beta}$. m, Bed 27F, $B_{II\gamma}$. N, Bed 33A, $B_{III\alpha}$. O, Bed K-4 marl, $B_{III\beta}$. P, Bed K+8 marl, $B_{III\gamma}$.

SI Materials and Methods

Preservational state of material and measures taken to identify samples affected by secondary diagenesis. Fe and Mn concentrations in biogenic carbonates are good indicators of diagenesis. These elements are efficiently removed from oxic seawater as clear from their short oceanic residence times[1] and they are incorporated only in very small amounts into the living shell of calcite shelled metazoans, such as brachiopods[2]. Because Fe and Mn are carried by anoxic pore waters and are compatible within the calcite crystal lattice[3], both elements are typically enriched in secondary calcite phases.

The cut off limit for good fossil preservation depends on the diagenetic context and should be chosen to avoid diagenetically overprinted materials as best as possible. Applied limits are therefore arbitrary to some degree. For the sections studied here, we adopted a limiting Mn/Ca ratio of 0.455 mmol/mol[4]. An uncontrollable amount of Fe was introduced during acid treatment of the samples analyzed for carbon and oxygen isotopes whereas iron concentrations could be reliably determined for samples analyzed for $^{87}Sr/^{86}Sr$ ratios. For the latter samples an upper limit for Fe/Ca ratios of 2 mmol/mol was adopted. Samples, for which one or both of these ratios exceeded the respective limit, were excluded from interpretation.

The efficiency of the cull is exemplified by comparison of geochemical data from samples termed "altered" with those showing a well-preserved geochemical signature. Altered samples exhibit a clear tendency to be depleted in Sr and ^{18}O and enriched in ^{87}Sr, whereas Mg/Ca ratios and especially $\delta^{13}C_{carb}$ values seem not to respond as strongly to alteration. These observations are perfectly compatible with previously published trends of alteration[5]. Additionally selected specimens were investigated using SEM to check for ultrastructure preservation (Supplementary Fig. 3). In general the shell structures were found to be well preserved, without any indications of secondary cements or pervasive recrystallization. Overall, the good correspondences between geochemical trends, shell preservation and diagenetic alteration provide good evidence that the paleoenvironmental information stored in the calcite shells are well-preserved in samples satisfying these geochemical tests.

Analytical details regarding the geochemical proxies. Geochemical analyses were carried out at the University of Copenhagen following published methods[6]. Carbon and oxygen isotope samples were prepared from brachiopods using stainless steel needles. The primary brachiopod shell layer and any visible impurities were removed and the foliate, secondary layers were targeted for sampling and analysis. Shell fragments were transferred into glass vials and dissolved with ~0.05 ml of >100% H_3PO_4 after flushing the vials with clean He for 4 minutes. After equilibration times of at least 100 min at a temperature of 70.0°C the resultant carbon dioxide was analyzed for $\delta^{13}C$ and $\delta^{18}O$, using an IsoPrime Gas Source Isotope Ratio Mass Spectrometer with a multiflow unit[7]. Weight dependent effects on the raw data were corrected for using a set of samples of the Copenhagen laboratory reference material (Carrara Marble: "LEO"), covering the weight range of the samples. The reproducibility (2 sd) of the analyses was found to be 0.08 ‰ for C and 0.18 ‰ for O isotope ratios.

The $^{87}Sr/^{86}Sr$ ratios were obtained using a Sector VG 54 Thermal Ionization Mass Spectrometer (TIMS). The sampled brachiopod shell material was dissolved in 0.2 M HNO_3 using 2 ml liquid per mg calcite. The resulting solution was homogenized by shaking and then a split of 1 ml was extracted for element ratio determination using ICP-OES. The remainder of the solution was transferred into a Teflon beaker, dried on a hotplate at 80°C and then re-dissolved in ~0.2 ml of 3 M nitric acid. The strontium was then purified using Sr-Spec resin[8], eluted with de-ionized water, and 25 µl of 0.1 M H_3PO_4 was added. The solution was dried on a hotplate at 80°C. Hereafter the material was loaded on single rhenium filaments in 5 µl of Ta_2O_5 H_3PO_4 matrix. $^{87}Sr/^{86}Sr$ ratios were measured on the TIMS with Faraday 8 cups in dynamic multi-collection mode at 1250 to 1400°C. Accuracy and reproducibility were checked with NISTSRM-987 which gave an average $^{87}Sr/^{86}Sr$ ratio of 0.710236 ± 0.000023 (2 sd, n = 17) in 2013. JLs-1 was taken for whole-procedure reproducibility control. 1 ml of a stock solution with Ca concentration ~1000 µg/g – equating to ~750ng of Sr – was processed using the same protocol as the samples, for which a similar or larger amount of Sr was processed. The average $^{87}Sr/^{86}Sr$ ratio for JLs-1 was found to be 0.707810 ± 0.000013 (n = 6).

Element ratios (Mg/Ca, Sr/Ca, Mn/Ca) were measured using a Perkin Elmer Optima 7000 DV ICP-OES on the reacted carbonate aliquots which remained from the H_3PO_4 treatment (Coleman et al., 1989). For samples prepared for $^{87}Sr/^{86}Sr$ ratio analysis, also the Fe/Ca ratio was determined. Samples were diluted with 2 % HNO_3 to a nominal Ca concentration of 25 µg/g and analyses conducted against a set of three matrix-matched, synthetic multi element solutions covering the expected compositional range of the calcite. Accuracy and reproducibility of the analyses were checked by multiple measurement of a stock solution of JLs-1 (split 1 - position 63-1) prepared from ~2 g powder reacted with 3 M HNO_3 (n = 114). Mg/Ca ratios (14.13 ± 0.2 mmol/mol, 2 sd) and Sr/Ca ratios (0.3416 ± 0.0058 mmol/mol, 2sd)

reproduce within 2 % (2 rsd), whereas Mn/Ca ratios of JLs-1 (0.0301 ± 0.0021, 2 sd) reproduce within 7 % (2 rsd) due to very low Mn concentrations. For Mn/Ca ratios above 0.1 mmol/mol reproducibility is generally better than 3 % (2 rsd)[6]. Measured average Mn/Ca and Sr/Ca ratios agree within 0.5 % with values published in the literature[9]. However, Mg/Ca ratios are measured 8 % lower than ratios computed from the literature.

References

1 Li, Y.-H. A brief discussion on the mean oceanic residence time of elements. *Geochimica et Cosmochimica Acta* **46**, 2671–2675 (1982).

2 Brand, U., Logan, A., Hiller, N. & Richardson, J. Geochemistry of modern brachiopods: applications and implications for oceanography and paleoceanography. *Chemical Geology* **198**, 305–334 (2003).

3 Rimstidt, J. D., Balog, A. & Webb, J. Distribution of trace elements between carbonate minerals and aqueous solutions. *Geochimica et Cosmochimica Acta* **62**, 1851–1863 (1998).

4 Korte, C., Jones, P. J., Brand, U., Mertmann, D. & Veizer, J. Oxygen isotope values from high-latitudes: Clues for Permian sea-surface temperature gradients and Late Palaeozoic deglaciation. *Palaeogeography, Palaeoclimatology, Palaeoecology* **269**, 1–16, doi:doi:10.1016/j.palaeo.2008.06.012. (2008).

5 Brand, U. & Veizer, J. Chemical diagenesis of a multicomponent carbonate system –2: stable isotopes. *Journal of Sedimentary Petrology* **51**, 987–997 (1981).

6 Ullmann, C. V. *et al.* Partial diagenetic overprint of Late Jurassic belemnites from New Zealand: implications for the preservation of δ^{7}Li values in calcite fossils. *Geochimica et Cosmochimica Acta* **120**, 80–96 (2013).

7 Spötl, C. & Vennemann, T. W. Continuous-flow isotope ratio mass spectrometric analysis of carbonate minerals *Rapid Communications in Mass Spectrometry* **17**, 1004–1006 (2003).

8 Horwitz, E. P., Chiarizia, R., Dietz, M.L. A novel strontium-selective extraction chromatographic resin. *Solvent Extraction and Ion Exchange* **10**, 313–336 (1992).

9 Imai, N., Terashima, S., Itoh, S. & Ando, A. 1996 compilation of analytical data on nine GSJ geochemical reference samples, "Sedimentary rock series". *Geostandards Newsletter* **20**, 165–216 (1996).

Supplementary Information Database (separate file)

This EXCEL spreadsheet presents the source data necessary to recreate and evaluate the various geochemical proxies used in our analysis. The subsheets presented here essentially duplicate the analysis of the geochemical proxies presented in our study. They are intended to benefit those who may want to reproduce, or, modify our observations in some way. This supplementary source data takes the form of 5 subsheets.
